# Tumor mutational burden in lung cancer: a systematic literature review

**DOI:** 10.18632/oncotarget.27287

**Published:** 2019-11-12

**Authors:** Connor Willis, Michelle Fiander, Dao Tran, Beata Korytowsky, John-Michael Thomas, Florencio Calderon, Teresa M. Zyczynski, Diana Brixner, David D. Stenehjem

**Affiliations:** ^1^ Department of Pharmacotherapy, University of Utah, Salt Lake City, UT, USA; ^2^ Department of Pharmacy Practice and Pharmaceutical Sciences, University of Minnesota, Duluth, MN, USA; ^3^ Bristol-Myers Squibb Company, Lawrenceville, NJ, USA

**Keywords:** biomarkers, chemotherapy, immunotherapy, lung cancer, PD-L1

## Abstract

**Purpose:** To assess the association of tumor mutational burden (TMB) with clinical outcomes, other biomarkers and patient/disease characteristics in patients receiving therapy for lung cancer.

**Results:** In total, 4,303 publications were identified; 81 publications were included. The majority of publications assessing clinical efficacy of immunotherapy reported an association with high TMB, particularly when assessing progression-free survival and objective response rate. High TMB was consistently associated with *TP53* alterations, and negatively associated with EGFR mutations. High TMB was also associated with smoking, squamous cell non-small cell lung carcinoma, and being male.

**Methods:** A systematic literature review based upon an a priori protocol was conducted following Preferred Reporting Items for Systematic Reviews and Meta-Analyses (PRISMA) and Cochrane methodologies. Searches were conducted in EMBASE, SCOPUS, Ovid MEDLINE^®^, and Emcare (from January 2012 until April 2018) and in two clinical trial registries. Conference abstracts were identified in EMBASE, and in targeted searches of recent major conference proceedings (from January 2016 until April 2018). Publications reporting data in patients receiving therapy for lung cancer that reported TMB and its association with clinical efficacy, or with other biomarkers or patient/disease characteristics, were included. Results are presented descriptively.

**Conclusion:** This systematic literature review identified several clinical outcomes, biomarkers, and patient/disease characteristics associated with high TMB, and highlights the need for standardized definitions and testing practices. Further studies using standardized methodology are required to inform treatment decisions.

## INTRODUCTION

The advent of immunotherapy has transformed the clinical oncology landscape in recent years, with significant improvements in long-term survival in some patients. However, a large proportion of patients do not respond to immunotherapies, and predictors of response are required to improve patient selection. Among investigated biomarkers, tumor mutational burden (TMB) has recently emerged as a potential predictor of response to immunotherapy in various tumor types [1, 2]. Increases in TMB are driven by several factors, including DNA replication errors mediated by defective tumor suppressor genes (e. g. *TP53)*, deficient DNA mismatch repair (dMMR) mechanisms (generally indicated by high microsatellite instability [MSI-H]), and exposure to mutagens such as tobacco, alkylating agents and ultraviolet light [3, 4]. Tumors with high levels of TMB are thought to express more cancer-specific antigens (neoantigens) that may sensitize them to immunotherapy [1, 5–7]. Accordingly, TMB levels have been shown to correlate with objective response rates (ORR) during immunotherapy across a number of cancer types [8].

Immunohistochemistry-determined programmed death ligand 1 (PD-L1) expression has been approved by the US Food and Drug Administration (FDA) as a companion diagnostic for several immunotherapies in various cancers [9]. In March 2015, the programmed cell death-1 (PD-1) inhibitor nivolumab was approved for second-line therapy of metastatic NSCLC independent of PD-L1 expression, as therapeutic response in phase 3 programs was largely independent of PD-L1 levels [10–12]. Conversely, in October 2015, the PD-1 inhibitor pembrolizumab received PD-L1-dependent FDA approval for second-line therapy of metastatic non-small cell lung cancer (NSCLC), based on observations that improved efficacy was associated with elevated PD-L1 expression [13]. Collectively, these studies reported that many patients with elevated PD-L1 levels did not respond to immunotherapy, while a substantial minority of patients who had low PD-L1 expression did experience clinical benefit [10–13]. These findings highlight the need for additional biomarkers to improve patient selection for these therapies.

Tumors with dMMR/MSI-H have demonstrated improved response rates to PD-1/PD-L1 inhibitors, and dMMR/MSI-H deficiency has become the first “tissue-agnostic” biomarker to receive FDA approval, for therapy with pembrolizumab [14]. However, the vast majority of tumor samples with high TMB do not exhibit dMMR/MSI-H [4], and non-dMMR/MSI-H deficient tumors may benefit from immunotherapy. Lung cancers are more frequently associated with high TMB compared with other cancer types [4, 15], and studies suggest an association between TMB and response to immunotherapy in patients with NSCLC [10, 16, 17].

Traditional evaluation of tissue TMB by whole exome sequencing (WES) using next-generation sequencing (NGS) technology has been expensive and labor-intensive [18]. Recent advances in comprehensive genomic profiling (CGP), using targeted NGS, which measures the number of mutations on a portion of the coding region while simultaneously providing data on specific DNA alterations, have been validated [19] and shown to reflect measurements obtained by WES [20]. Based on parallel FDA approval, the Centers for Medicare & Medicaid Services proposed coverage for the FoundationOne CDx *in vitro* diagnostic test for NGS evaluation of gene mutations in solid tumors [21]. These developments have made routine TMB evaluation increasingly feasible. Nevertheless, the methods of reporting TMB in lung cancer remain highly inconsistent; some studies report TMB in terms of the absolute number of mutations, while others assess mutations per DNA megabase (mut/Mb). Additionally, thresholds used to denote high TMB vary greatly and no widely used standard currently exists.

This is the first systematic literature review describing the role of TMB as a predictive biomarker in patients with lung cancer. We aimed to assess associations between TMB and clinical efficacy outcomes in patients receiving therapy for lung cancer, to identify other biomarkers related to TMB, and to understand the association of patient and disease characteristics with TMB. Additionally, we sought to describe how TMB testing is implemented in clinical practice and reported in the literature.

## RESULTS

### Publication screening

**Figure 1:**
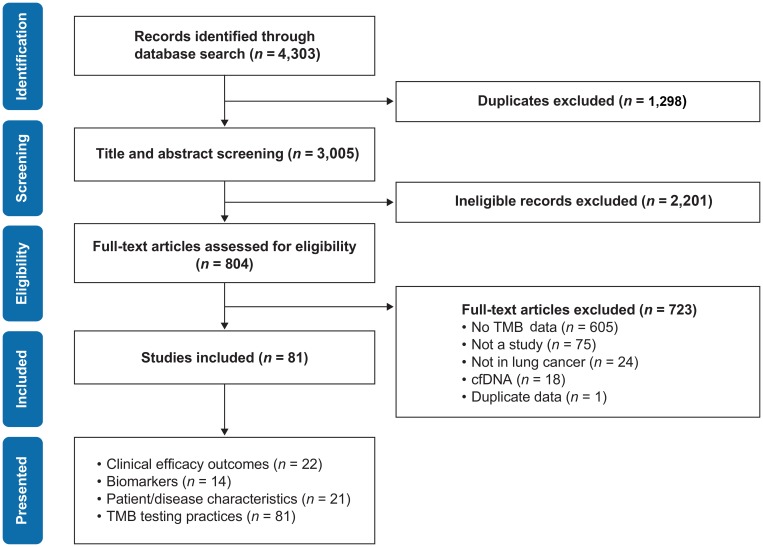
PRISMA flow diagram of publication identification process. TMB, tumor mutational burden. ‘Presented’ categories are not mutually exclusive.

Searches retrieved 4,303 publications in total, of which 1,298 were duplicates, 2,201 were excluded based on titles and abstracts, and 723 were excluded based on full-text; 81 studies were included (Figure 1). Most articles identified were published in 2017 (54 publications; 65.9%), with 16 (19.5%) published from January to April 2018. A summary of relevant data reported by publications presenting efficacy outcomes, biomarkers and/or patient or disease characteristics is presented in Supplementary Table 4.

### Clinical efficacy outcomes and TMB

In total, 22 publications presenting data on TMB also reported one or more clinical outcomes (Table 1) [1, 10, 16, 27–45]. The results of our risk of bias analysis are summarized in Supplementary Figure 1. Of these, one publication reported on patients with small cell lung cancer (SCLC) [32], one reported on a combination of NSCLC and SCLC [36], and the rest reported on patients with NSCLC. Of the 22 publications that reported clinical outcomes, 14 used CGP to assess TMB [16, 28, 30, 33, 34, 36–41, 43–45] while eight studies used WES [1, 10, 27, 29, 31, 32, 35, 42]. Of the 13 publications assessing TMB using CGP, six used a Foundation Medicine platform [16, 28, 30, 33, 41, 45], three used a combination of commercial platforms (including Foundation Medicine, Guardant360, Caris Life Sciences, and Precipio) [37, 40, 44], one used only Caris platform results [38], one used only MSK-IMPACT results [34], and three did not specify which testing platforms were used [36, 39, 43].

**Table 1:** Summary of characteristics of publications reporting efficacy data

Publication	Lung cancer type	TMB threshold values	Agent/Setting	TMB-H patients/total patients assessed for TMB (*n*/*N*)
Carbone 2017 [10]	NSCLC	TMB-L: < 100 mutations TMB-I: 100–242 mutations TMB-H: > 242 mutations	Nivolumab/1L Platinum-based chemotherapy	47/158 60/154
Choi 2017 [27]	NSCLC	NR	NR	NR/108
Davis 2017 [28]	NSCLC	TMB-L: < 15 mut/Mb TMB-H: ≥ 15 mut/Mb	PD-1/PD-L1 inhibitors	NR/35
Gettinger 2017 [29]	NSCLC	NR	PD-1/PD-L1 inhibitors	NR/45
Goodman 2017 [16]	NSCLC	TMB-L: 1–5 mut/Mb TMB-I: 6–19 mut/Mb TMB-H: ≥ 20 mut/Mb	PD-1/PD-L1 inhibitors	3/36
Haratani 2017 [31]	NSCLC	NR	Nivolumab	NR/9
Hellmann 2017 [34]	NSCLC	TMB-L: Below 85th percentile TMB-H: Above 85th percentile	PD-L1 inhibitors +/- anti-CTLA-4 therapy	NR/437
Hellmann 2018 [33]	NSCLC	TMB-L: < 10 mut/Mb TMB-H: ≥ 10 mut/Mb	Nivolumab + ipilimumab Chemotherapy	139/330 160/349
Hellmann 2018 [32]	SCLC	TMB-L: < 143 mutations TMB-I: 143–247 mutations TMB-H: > 247 mutations	Nivolumab Nivolumab + ipilimumab	47/133 26/78
Hu 2018 [35]	NSCLC	TMB-H: ≥ 20 mut/Mb	PD-1/PD-L1 inhibitors	9/NR
Kowanetz 2017 [30]	NSCLC	TMB-L: Below 50th percentile TMB-H: Above 50th percentile	Atezolizumab/1L Atezolizumab/2L	NR/102 NR/371
Mahadevan 2017 [36]	NSCLC (*n* = 80) and SCLC (*n* = 5)	TMB-L: Below 50th percentile TMB-H: Above 50th percentile	PD-1 (*n* = 82)/ PD-L1 (*n* = 5) inhibitors/ other agents (*n* = 7)	NR/94
Park 2017 [37]	NSCLC	TMB-L: 1–5 mut/Mb TMB-I: 6–19 mut/Mb TMB-H: > 20 mut/Mb	Nivolumab	NR/36
Patel 2017 [38]	NSCLC	NR	Immunotherapy	NR/50
Rizvi 2015 [1]	NSCLC	TMB-L: Below 50th percentile TMB-H: Above 50th percentile	Pembrolizumab (cohort 1) Pembrolizumab (cohort 2)	NR/16 NR/18
Ross 2017 [45]	NSCLC	NR	Immune checkpoint inhibitors	545/3758
Roszik 2016 [39]	NSCLC	TMB-L: < 100 mutations TMB-H: ≥ 100 mutations	Pembrolizumab	21/29
Rozenblum 2017 [40]	NSCLC	NR	Pembrolizumab and nivolumab	NR/18
Singal 2017 [41]	NSCLC	TMB-L: 1–5 mut/Mb TMB-I: 6–19 mut/Mb TMB-H: ≥ 20 mut/Mb	Nivolumab	NR/444
Wang 2017 [42]	NSCLC	TMB-L: Below 50th percentile TMB-H: Above 50th percentile	NR	NR/98
Xiao 2016 [43]	NSCLC	TMB-L: ≤ 4 mutations TMB-H: > 4 mutations	NR	47/335
Yaghmour 2016 [44]	NSCLC	TMB-L: Below 80th percentile TMB-H: Above 80th percentile	Nivolumab, pembrolizumab, or ipilimumab	3/23

Abbreviations: 1L, first-line; CTLA-4, cytotoxic T-lymphocyte-associated protein 4; mut/Mb, mutations per DNA megabase; NR, not reported; NSCLC, non-small cell lung cancer; PD-1, programmed cell death-1; PD-L1, programmed death ligand 1; SCLC, small cell lung cancer; TMB, tumor mutational burden; TMB-H, high TMB; TMB-I, intermediate TMB; TMB-L, low TMB.

### Overall survival

Twelve publications presented data on overall survival (OS) and TMB (Table 2) [10, 16, 27–30, 32, 37, 38, 41, 43, 44]. Of these, ten publications assessed patients receiving immunotherapies [10, 16, 28–30, 32, 37, 38, 41, 44], with two assessing more than one treatment arm [30, 32]. Eight of these (including the one study assessing SCLC) reported longer OS in patients with high TMB compared with low TMB [10, 16, 28, 30, 32, 37, 41, 44]; yet only two reported statistical significance [28, 41]. Two publications reported a non-significant association between TMB and OS during immunotherapy, but provided no details on the definition of high TMB and did not present median OS [29, 38]. Two further publications presented data for OS and TMB but did not specify the type of treatment used [27, 43]; one of these reported that OS was significantly longer in patients with low TMB compared with high TMB [43], with the other publication reporting no significant difference [27].

**Table 2:** Publications reporting primary efficacy outcomes (OS, PFS, ORR) in patients receiving active therapy for lung cancer

	Median OS (Months) [HR (95% CI)/*P* Value]	Median PFS (Months) [HR (95% CI)/*P* Value]	ORR (%) [*P* Value]
**IMMUNOTHERAPY**			
Carbone 2017 [10]			
Nivolumab/1L	Low/intermediate vs high TMB: 12.7 vs 18.3 [*P*: NR]	Low vs intermediate vs high TMB: 6.9 vs 6.5 vs 9.7 [*P*: NR]	Low/intermediate vs high TMB: 23% vs 47% [*P*: NR]
Davis 2017 [28]			
PD-1/PD-L1 inhibitors	Longer with high TMB [0.19 (0.04 to 0.88); *P* = .034]	NR	NR
Gettinger 2017 [29]			
PD-1/PD-L1 inhibitors	NR [*P* = .92]	NR	Higher with high TMB [*P* = .02]
Goodman 2017 [16]			
PD-1/PD-L1 inhibitors	Low/intermediate vs high TMB: 7.6 vs not reached [0.32 (0.07 to 1.50); *P* = .15]	Low/intermediate vs high TMB: 2.1 vs 12.5 [0.32 (0.13 to 0.81); *P* = .0817]	Low/intermediate vs high TMB: 18% vs 33% [*P* = .4882]
Kowanetz 2017 [30]			
Atezolizumab/1L	Longer with high TMB [50th percentile: 0.79 (0.39 to 1.58), *P*: NR; 75th percentile: 0.45 (0.17 to 1.16), *P*: NR]	Longer with high TMB [50th percentile: 0.58 (0.36 to 0.94), *P*: NR; 75th percentile: 0.54 (0.3 to 0.97), *P*: NR]	Low vs high TMB (50th percentile): 13% vs 28% [*P*: NR] Low vs high TMB (75th percentile): 20% vs 25% [*P*: NR]
Atezolizumab/2L	Longer with high TMB [50th percentile: 0.87 (0.65 to 1.16), *P*: NR; 75th percentile: 0.7 (0.49 to 1.0), *P*: NR]	Longer with high TMB [50th percentile: 0.64 (0.5 to 0.8), *P*: NR; 75th percentile: 0.5 (0.38 to 0.67), *P*: NR]	Low vs high TMB (50th percentile): 14% vs 25% [*P*: NR] Low vs high TMB (75th percentile): 16% vs 29% [*P*: NR]
Haratani 2017 [31]			
Nivolumab	NR	NR	Higher with high TMB [*P* = .038]
Hellmann 2017 [34]			
Anti-PD-L1 +/- anti-CTLA-4 therapy	NR	Longer with high TMB [HR: 0.59 (95% CI: NR); *P* = .004]	NR
Hellmann 2018 [33]			
Nivolumab + Ipilimumab	NR	Low vs high TMB: 3.2 vs 7.2 [*P*: NR]	NR
Hellmann 2018 [32]			
Nivolumab	Low/intermediate vs high TMB: 3.1 vs 5.4 [*P*: NR]	Low/intermediate vs high TMB: 1.3 vs 1.4 [*P*: NR]	Low/intermediate vs high TMB: 7% vs 21% [*P*: NR]
Nivolumab + ipilimumab	Intermediate vs high TMB: 3.4 vs 22.0 [*P*: NR]	Low/intermediate vs high TMB: 1.3 vs 7.8 [*P*: NR]	Low/intermediate vs high TMB: 22% vs 46% [*P*: NR]
Mahadevan 2017 [36]			
PD-1/PD-L1 inhibitors	NR	Longer with high TMB [*P* = . 015]	NR
Park 2017 [37]			
Nivolumab	Low vs intermediate vs high TMB: 12.4 vs 10.3 vs not reached [*P* = .211]	NR	NR
Patel 2017 [38]			
Immunotherapy	NR [*P = .*5]	NR	NR
Rizvi 2015 [1]			
Pembrolizumab (cohort 1)	NR	Low/intermediate vs high TMB: 3.7 vs 14.5 [*P* = .01]	Low vs high TMB: 0% vs 63% [*P* = .03]
Pembrolizumab (cohort 2)	NR	Low/intermediate vs high TMB: 3.4 vs not reached [*P* = .006]	Low vs high TMB: 22% vs 56% [NR]
Roszik 2016 [39]			
Pembrolizumab	NR	Low vs high TMB: 4.1 vs 8.3 [*P* = .0003]	Low vs high TMB: 0% vs 48% [NR]
Singal 2017 [41]			
Nivolumab	Low/intermediate vs high TMB: 10 vs not reached [*P* < .01]	NR	NR
Yaghmour 2016 [44]			
Nivolumab, pembrolizumab, or ipilimumab	Low vs high TMB: 4.9 vs not reached [HR undefined (95% CI: 0.04 to 1.90); *P = .*21]	NR	NR
**CHEMOTHERAPY**			
Carbone 2017 [10]			
Platinum-based chemotherapy	NR	Low vs intermediate vs high TMB: 4.2 vs 3.6 vs 5.8 [NR]	Low/intermediate vs high TMB: 33% vs 28% [NR]
Hellmann 2018 [33]			
Chemotherapy	NR	Low vs high TMB: 5.5 vs 5.5 [NR]	NR
**NOT SPECIFIED**			
Choi 2017 [27]			
Not specified	NR [*P* = .5933]	NR [*P* = .7765]	NR
Wang 2017^*^ [42]			
Not specified	NR	Shorter with high TMB^*^ [*P* = .0133]	NR
Xiao 2016 [43]			
Not specified	Low vs high TMB: 61 vs 48.4 [*P* = .02]	NR	NR

Abbreviations: CI, confidence interval; HR, hazard ration; NR, not reported; ORR, objective response rate; OS, overall survival; PFS, progression-free survival; TMB, tumor mutational burden.

^*^Wang 2017 presents disease-free survival, not progression-free survival.

### Progression-free survival

Eleven publications presented data for progression-free survival (PFS) and TMB (Table 2) [1, 10, 16, 27, 30, 32–34, 36, 39, 42]. Of these, nine publications (including the one SCLC study) assessed patients receiving immunotherapies [1, 10, 16, 30, 32–34, 36, 39], with all reporting longer PFS in patients with high TMB compared with low TMB. Six of these publications also indicated that the difference in PFS was significant, either by hazard ratio confidence intervals (CIs) less than 1, or by *P* value [1, 16, 30, 34, 36, 39]. Two publications presented data for PFS and TMB in patients receiving chemotherapy, with equivocal differences in PFS and no statistical comparison presented [10, 33]. Two further publications did not specify the type of treatment received [27, 42]; one of these reported that PFS was significantly shorter in patients with high TMB compared with low TMB [42], with the other reporting no significant difference [27].

### Objective response rate

Eight publications presented data for ORR and TMB in patients receiving immunotherapies (including the one SCLC study) (Table 2) [1, 10, 16, 29–32, 39]; all reported higher ORR in patients with high TMB compared with low TMB. Three of these publications reported statistically significant differences [1, 29, 31], with another reporting no significant difference [16]. The other four publications did not present statistical comparisons. One publication also presented data for ORR and TMB in patients receiving platinum-based chemotherapy; there appeared to be no association of TMB on ORR in these patients [10].

### Durable clinical benefit

Four publications presented data on durable clinical benefit (DCB) and TMB in patients receiving immunotherapies (Supplementary Table 5) [1, 34, 36, 39]. Three reported that TMB was significantly higher in patients who experienced DCB compared with those who did not [1, 34, 36]. One further publication reported patients with high TMB were more likely to experience DCB than patients with low TMB, but did not present statistical comparisons [39].

### Disease control

Two studies presented data on disease control rate (DCR) and TMB in patients receiving immunotherapy (Supplementary Table 5) [35, 41]. One of these reported that DCR was significantly higher in patients with high TMB compared with patients with low TMB [41]. The other publication included only patients with high TMB, but reported DCR of 100% [35].

An additional publication reported no clear association of TMB levels with response as determined by Response Evaluation Criteria In Solid Tumors (RECIST) criteria (partial response, stable disease, or progressive disease) in patients receiving immunotherapies (Supplementary Table 5) [40].

### Duration of treatment

Two publications compared duration of therapy (DoT) with immunotherapy in patients with high and low TMB; both of these reported that DoT was significantly longer in patients with high TMB (Supplementary Table 5) [41, 45].

### Biomarkers and TMB

A total of 14 publications reporting the association of TMB with biomarkers were identified (Table 3) [10, 27, 36, 45–55]. Nine publications reported data on PD-L1 and TMB [10, 27, 36, 45, 48–50, 53, 55]. Four reported significant associations of PD-L1 and TMB [45, 48, 53, 55], with two reporting weak Spearman’s correlations of 0.12 and 0.085 [45, 48]. Three publications (one of which presented three analyses from different centers) assessed TMB and EGFR status; all reported that EGFR-mut was significantly associated with low TMB [46, 47, 51]. All three publications assessing *TP53* alterations reported that it was significantly associated with high TMB [51, 52, 54].

**Table 3:** Publications reporting TMB and relevant biomarkers

Publication	Main findings relating to TMB and biomarker	*P* Value
**PD-1/PD-L1**		
Carbone 2017 [10]	Pearson’s r: 0.059	NA
Choi 2017 [27]	Pearson’s r:	
	PD-1: 0.004	PD-1: .96958
	PD-L1: –0.067	PD-L1: .52563
Goldberg 2017 [48]	Spearman’s rho: 0.12	.00035
Liu 2018 [49]	Spearman’s rho: 0.092	.62
Mahadevan 2017 [36]	Undefined	.47
Nakagomi 2018 [50]	Undefined	.49
Schabath 2017 [53]	Undefined	.03
Senarathne 2018 [55]	Undefined	.05
Ross 2017 [45]	Spearman’s rho: 0.085	.00062
**EGFR**		
Chen 2017 [46]	Median TMB higher in EGFR-wt patients (8.4) vs EGFR-mut patients (4.6)	.034
Dong 2017 [47]		
TCGA	Median TMB higher in EGFR-wt patients (181) vs EGFR-mut patients (56)	< .001
Broad Institute	Median TMB higher in EGFR-wt patients (209) vs EGFR-mut patients (59)	.003
Guangdong Lung Cancer Institute	Median TMB higher in EGFR-wt patients (197) vs EGFR-mut patients (162)	.029
Owada 2017 [51]	Median TMB higher in EGFR-wt patients vs EGFR-mut patients (values NR)	< .001
***TP53***		
Owada 2017 [51]	Median TMB higher in *TP53*-positive patients vs *TP53*-negative patients (values NR)	< .001
Rothberg 2017 [52]	Median TMB higher in *TP53*-positive patients vs *TP53*-negative patients (values NR)	< .0001
Schrock 2017 [54]	Median TMB higher in *TP53*-positive patients (10.1) vs *TP53*-negative patients (5)	.001

Abbreviations: NR, not reported; PD-1, programmed cell death-1; PD-L1, programmed death ligand 1; TMB, tumor mutational burden.

### Patient/disease characteristics and TMB

We identified 22 publications that evaluated the association between patient or disease characteristics and TMB (Table 4). Smoking history was the most commonly reported; of the 16 publications presenting data on smoking history and TMB [1, 28, 36, 42, 43, 51, 56–65], 14 reported that smoking was significantly associated with higher TMB levels [1, 36, 42, 43, 51, 57, 59–62, 64, 65] or with a greater likelihood of a patient having high TMB [28, 56]. Two further publications assessed TMB levels in patients with a history of smoking; one did not assess significance [63], and the other reported non-significance [58].

**Table 4:** Publications reporting data on key patient and disease characteristics and TMB

Publication	Main findings relating to TMB and patient/disease characteristic	*P* Value
**SMOKING**		
Kadara 2017 [57]	TMB was higher in patients with a history of smoking vs no smoking history (values NR)	.002
Kim 2017 [58]	TMB was higher in patients with a history of smoking (101.5 mutations) vs no smoking history (63.0 mutations)	.43
Mahadevan 2017 [36]	TMB was higher in patients with a history of smoking vs no smoking history (values NR)	.047
Ono 2017 [60]	TMB was higher in patients with a history of smoking vs no smoking history (values NR)	.0001
Owada 2017 [51]	TMB was higher in patients with a history of smoking vs no smoking history (values NR)	< .001
Quek 2018 [61]	TMB was higher in patients with a history of smoking (11.6 mut/Mb) vs no smoking history (4.0 mut/Mb)	.00016
Reck 2017 [62]	TMB was higher in patients with a history of smoking (199 mutations) vs no smoking history (60 mutations)	.004
Rizvi 2015 [1]	TMB was higher in patients with a history of smoking vs no smoking history (values NR)	.08
Schrock 2016 [63]	TMB was higher in patients with a history of smoking (10.4 mut/Mb) vs no smoking history (3.3 mut/Mb)	NR
Shim 2015 [64]	TMB was higher in patients with a history of smoking vs no smoking history (values NR)	< .0001
Wang 2017 [42]	TMB was higher in patients with a history of smoking vs no smoking history (values NR)	.00087
Xiao 2016 [43]	TMB was higher in patients with a history of smoking (3 mut/Mb) vs no smoking history (2 mut/Mb)	.00139
Xiao 2017 [65]	TMB was higher in patients with a history of smoking (126 mutations) vs no smoking history (46 mutations)	.031
Chae 2018 [56]	Patients with a history of smoking were more likely to have TMB-H than TMB-L (values NR)	< .001
Davis 2017 [28]	Smoking was associated with higher TMB mutation (TMB-H: 18 patients with history of smoking vs 1 patient with no history; TMB-L: 38 patients with history of smoking vs 25 patient with no history)	.001
Lizotte 2016 [59]	Spearman’s rho: 0.5297	.0009
**HISTOLOGY**		
Isaka 2017 [66]	TMB higher in patients with SCC (5.6 mut/Mb) vs adenocarcinoma (1.6)	NR
Kojima 2017 [67]	TMB higher in patients with SCC (5.6 mut/Mb) vs adenocarcinoma (1.6)	NR
Ono 2017 [60]	TMB higher in patients with SCC vs adenocarcinoma (no values reported)	.069
Owada 2017 [51]	TMB higher in patients with SCC vs adenocarcinoma (no values reported)	< .001
Schrock 2017 [54]	TMB higher in patients with non-adenocarcinoma (9.9 mut/Mb) vs adenocarcinoma (6.8 mut/Mb)	.176
**SEX**		
Owada 2017 [51]	TMB higher in male patients vs female (values NR)	< .001
Xiao 2016 [43]	TMB higher in male patients (3 mut/Mb) vs female (2 mut/Mb)	< .001
Xiao 2017 [65]	TMB higher in male patients (92 mutations) vs female (34 mutations)	< .001
**CANCER STAGE**		
Choi 2017 [27]	No association of disease stage and TMB in patients with stage I–III cancer (values NR)	.95
Kim 2018 [58]	TMB higher in patients with stage I–III cancer (104.4 mutations) vs stage IV (80.0 mutations)	.277
Xiao 2016 [43]	No clear differences in TMB between patients with stage I (2 mutations), stage II (2 mutations), stage III (2 mutations) and stage IV (2 mutations)	NR
Xiao 2017 [65]	No clear differences in TMB between patients with stage I (39.5 mutations), stage II (74.5 mutations), stage III (59.0 mutations) and stage IV (50.5 mutations)	NR
**AGE**		
Ono 2017 [60]	TMB higher in patients aged ≥ 70 years vs < 70 years (values NR)	.106
Wang 2017 [42]	TMB higher in patients aged ≥ 65 years vs < 65 years (values NR)	.0208
Xiao 2016 [43]	No difference in TMB in patients aged ≥ 65 years (2 mutations) vs <65 years (2 mutations)	.616
Xiao 2017 [65]	TMB higher in patients aged ≥ 65 years (80.5 mutations) vs < 65 years (48 mutations)	.897
Zhang 2016 [68]	Log-2 transformed effect estimates for age in patient subgroups:	
	Adenocarcinoma *TP53*-positive: –0.023	.007
	Adenocarcinoma *TP53*-negative: 0.01	.30
	Squamous cell *TP53*-positive: –0.009	.22
	Squamous cell *TP53*-negative: –0.011	.37

Abbreviations: mut/MB, mutations per DNA megabase; NR, not reported; SCC, squamous cell carcinoma; TMB, total mutational burden.

Five publications presented data on TMB in NSCLC stratified by squamous or adenocarcinoma histology (Table 4) [51, 54, 60, 66, 67]. All reported higher TMB in patients with squamous cell or non-adenocarcinoma compared with patients with adenocarcinoma [51, 60, 66, 67]; however, only one demonstrated a significant difference [51]. Four publications assessed lung cancer stage, with all reporting no significant association with TMB [27, 43, 58, 65].

### Demographics

All three publications assessing TMB and sex reported that men had significantly higher TMB than women (Table 4) [43, 51, 65]. Four studies [42, 43, 60, 65] assessed age and TMB. Three reported higher TMB in older patients [42, 60, 65], with one demonstrating significance [42]. One publication investigated age in four subgroups across *TP53* status and cancer histology [68]. This study reported a small, but significant, inverse association of age and TMB in patients with *TP53*-mut adenocarcinoma, but no significant effect of age and TMB in patients with *TP53-*wt adenocarcinoma, or with *TP53-*wt or *TP53*-mut squamous cell carcinoma.

### TMB testing practices

A summary of the TMB testing practices is presented in Table 5. Among the 81 publications identified that reported TMB in lung cancer, 37 (46%) reported TMB as mut/Mb [16, 28, 30, 33–35, 37, 40, 41, 45, 46, 48, 54, 60, 61, 63, 66, 67, 69–87], 34 (42%) reported TMB as total mutations [1, 10, 32, 36, 38, 39, 42–44, 47, 49–51, 53, 55–57, 59, 62, 64, 65, 68, 88–99], and two reported both mut/MB and total mutations [31, 100]; eight publications did not report TMB in either mut/MB or total mutations [27, 29, 52, 101–105].

**Table 5:** Summary of reporting of TMB testing practices reported by 81 included publications

**Genes tested, *n* (%)**	27 (33%) [10, 30, 32–36, 39–41, 44, 46, 48, 55, 63, 70, 71, 73–75, 86, 87, 91, 94, 100, 101, 104]
Median (range) reported values	315 (15–592)
**Mbs tested, *n* (%)**	10 (12%) [16, 33, 40, 45, 54, 63, 70, 71, 81, 84]
Median (range) reported values	1.1 (0.8–1.2)
**Units used for TMB measurement, *n* (%)**	
Mut/Mb	37 (46%) [16, 28, 30, 33–35, 37, 40, 41, 45, 46, 48, 54, 60, 61, 63, 66, 67, 69–87]
Total mutations	34 (42%) [1, 10, 32, 36, 38, 39, 42–44, 47, 49–51, 53, 55–57, 59, 62, 64, 65, 68, 88–99]
Both	2 (2%) [31, 100]
Other units	8 (10%) [27, 29, 52, 101–105]
**TMB threshold defined, *n* (%)**	33 (41%) [1, 10, 16, 28, 30, 32–37, 39, 41–44, 48, 51, 54–56, 60, 63, 66, 69–71, 75, 84, 86, 91, 99, 102]
By mut/Mb	16 (48%) [16, 28, 33, 35, 37, 41, 48, 54, 63, 66, 69–71, 75, 84, 91]
Median (range) reported values	20 (10–20)
By percentiles	10 (30%) [1, 30, 34, 36, 44, 51, 56, 60, 86, 99]
Median (range) reported values	50 (50–85)
By total mutations	6 (18%) [10, 32, 39, 42, 43, 55]
Range of reported values	4–242

Abbreviations: mut/Mb, mutations per DNA megabase; TMB, tumor mutational burden.

In total, 33 publications (41%) classified TMB by high versus low or intermediate levels [1, 10, 16, 28, 30, 32–37, 39, 41–44, 48, 51, 54–56, 60, 63, 66, 69–71, 75, 84, 86, 91, 99, 102]. However, among these publications, various units were used to define the threshold of high or low/intermediate TMB: 16 publications (48%) classified TMB by mut/Mb (median threshold [range]: 20 [10–20]) [16, 28, 33, 35, 37, 41, 48, 54, 63, 66, 69–71, 75, 84, 91], 10 publications (30%) used a percentile to categorize TMB status [1, 30, 34, 36, 44, 51, 56, 60, 86, 99], and six publications (18%) categorized TMB based on total mutation count (range: 4–242) [10, 32, 39, 42, 43, 55].

## MATERIALS AND METHODS

A systematic literature review based on an a priori protocol (available upon request) was conducted, following Preferred Reporting Items for Systematic Reviews and Meta-Analyses (PRISMA) [22] and Cochrane Handbook for Systematic Reviews of Interventions [23] guidance.

### Literature search/data sources

Search strategies combined keywords and controlled vocabulary; full search strategy details are presented in Supplementary Tables 1–3. Bibliographic databases (EMBASE, Scopus, Ovid MEDLINE^®^ and Ovid Emcare) were searched from January 2012 until April 2018 for publications in the English language only. Methodological filters for both randomized controlled trials and observational studies were used. Both ClinicalTrials. gov and the International Clinical Trials Registry Platform were searched from January 2016 until April 2018 for ongoing, completed and in-progress studies. Conference abstracts were identified in Embase, and from review of the 2016–2018 conference proceedings for the American Society of Clinical Oncology (ASCO), European Society for Medical Oncology (ESMO), Society for Immunotherapy of Cancer (SITC), International Association for the Study of Lung Cancer (IASLC), and American Association for Cancer Research (AACR). Reference lists of included studies and related reviews were scanned for references missed by other methods of searching.

### Publication inclusion criteria

Publications reporting controlled or observational studies (excluding case reports and case series) in adults receiving therapy for any stage or histology of lung cancer were included. Publications that evaluated TMB solely by liquid biopsy were excluded, as this method has not yet been fully validated and evidence is new and emerging [24]; however, publications reporting results from both tissue and liquid biopsies were included. Definitions of “low”, “intermediate” and “high” levels of TMB were based on those used in the publications. Studies were included if they evaluated the association of TMB with clinical efficacy outcomes, biomarkers, or patient and disease characteristics. Clinical efficacy outcomes of interest included OS, PFS, ORR, DCB, DCR, and DoT. Biomarkers included PD-L1 expression, MSI status, MMR pathway gene mutations, DNA polymerase gene mutations, and any other genomic alterations. Clinical and demographic patient characteristics included sex, age, smoking status, cancer stage, and histology. Results for biomarkers and patient/disease characteristics are presented only for outcomes reported by at least three publications. Reported statistical comparisons were considered significant only when *P* < .05.

### Screening, study selection, and data extraction

Screening of search results was performed using Covidence [25], and was conducted independently by two reviewers. Search results were initially screened for eligibility based on review of titles and abstracts. After initial exclusion of publications that clearly did not meet inclusion criteria based on screening review of title and abstract, the full text of each remaining publication was reviewed. Data were extracted independently by two reviewers using Covidence. Disagreements were resolved by discussion or by a third reviewer.

### Evaluation of study quality and publication bias

Risk of bias was assessed using the Criteria for Critically Appraising Studies of Prognostic Tests for studies reporting clinical outcomes [26]. This method was adopted owing to the data reported and the purpose of the review. Bias was considered based on the following questions: “*Was an inception cohort assembled?*”; “*Was the referral pattern described?*”; “*Were laboratory and clinical outcomes assessed in a blinded fashion?”*; “*Was complete follow-up achieved?”*; “W*as adjustment for extraneous prognostic factors carried out?”*; “*Were appropriate statistical methods used?”* Two reviewers graded each study as low, high, or unclear risk of bias for each of the study design features, with discrepancies resolved by a third reviewer.

## DISCUSSION

Despite inconsistencies in the definitions and reporting of TMB, high TMB appeared to be associated with greater clinical benefit (particularly ORR and PFS) among patients receiving immunotherapy for lung cancer. This suggests that TMB, in addition to PD-L1 expression level and dMMR/MSI-H status, may have clinical utility in identifying patients likely to respond to immunotherapies. It should be noted that almost all publications included in our clinical efficacy analysis reported exclusively on patients with NSCLC, with one assessing a cohort in which 94% of patients had NSCLC [36], and one reporting on patients with SCLC [32]. Clinical outcomes in the study assessing patients with SCLC were qualitatively similar to the broader NSCLC findings, with improved OS, PFS, and ORR in patients with high TMB. These findings are supported by recent observations from the CheckMate 227 study, the first phase 3 trial in lung cancer to assess immunotherapy specifically in patients with high TMB (≥ 10 mut/Mb) [33]. That study reported that in patients receiving first-line therapy for NSCLC with high TMB, PFS was longer with nivolumab plus ipilimumab than with chemotherapy irrespective of PD-L1 levels (7.2 months vs 5.5 months; hazard ratio for disease progression or death [95% CI]: 0.58 [0.41 to 0.81]). Other trials assessing the predictive utility of TMB as a marker of response to immunotherapy in lung cancer are underway (NCT02848651; NCT03668119).

Evidence for an association between PD-L1 expression and TMB was inconsistent. This is not entirely unexpected, as previous studies have reported that PD-L1 and TMB are independent predictors of response to immunotherapy in patients with NSCLC [10, 33]. One retrospective analysis has reported that patients with both positive PD-L1 expression (defined as ≥ 1% expression) and high TMB had significantly improved ORR and PFS compared with patients who lacked one or both of these biomarkers [106]. Additional research is required to fully elucidate the clinical utility of TMB in combination with PD-L1 [107].

Our review found that TMB was consistently associated with *TP53* alterations. Somatic alteration in the *TP53* gene, which functions as a tumor suppressor in response to mutagenic cellular stresses, is one of the most frequent alterations in cancers, including lung cancers, although its predictive and/or prognostic role remains unclear [108]. Conversely, we found that TMB was negatively associated with EGFR mutations. In patients with EGFR-mut lung cancer, TMB has been shown to be associated with a poor response to tyrosine kinase inhibitors [109], and a meta-analysis of clinical trial data reported that patients with EGRF-mut tumors demonstrate a poor therapeutic response to immunotherapies [110]. Recent work has been designed to elucidate the interaction between these biomarkers, with one meta-analysis reporting that concurrent *TP53* and EGFR mutations predict poor response to tyrosine kinase inhibitors, but not to non-targeted therapies [111]. The precise nature of this interaction, and the involvement of TMB, remain unclear.

Several publications reported significant associations between TMB and multiple patient and disease characteristics, including a history of smoking, squamous cell lung carcinoma, and male sex. Of particular note, an association between smoking and high TMB levels was repeatedly demonstrated. This consistent finding is in line with previous observations that cancers related to chronic mutagenic exposures (such as from tobacco) exhibit the highest prevalence of mutations [15]. Interestingly, subgroup analyses of NSCLC patients receiving second-line nivolumab in the CheckMate 057 and 017 studies also found history of smoking to be associated with efficacy [11, 12]. Collectively, these characteristics may help identify patients who are more likely to have high TMB and therefore to respond to immunotherapy.

Limitations of this study included the diversity of testing methods and reporting of TMB. A standardized threshold for classifying TMB levels as low, intermediate, and high does not currently exist; and in the identified publications not only were the units used to report TMB inconsistent, but even publications reporting TMB in identical units often used different threshold definitions. Furthermore, testing platforms differ, such as between commercial and institutional sites, with varying institutional guidance regarding the number of genes and/or Mb to be tested, as well as the units used to report TMB findings. Additionally, although the majority of included data comes from solid tissue biopsies, some TMB data from evaluation of peripheral blood samples are also included. Future research is needed to fully evaluate the use of blood samples to assess TMB, and to compare these findings with TMB results from paired solid tumor samples. These inconsistencies impede any meaningful meta-analysis of the collated data. Other limitations of this study include the heterogeneity of lung cancer type and treatment regimens used. Consideration must also be given to the fact that our search included only a few high-impact congresses and may have overlooked relevant data reported elsewhere.

As most identified studies assessed TMB as a secondary/exploratory outcome, statistical power and reporting detail were generally insufficient. As a result, evidence of TMB as a predictive biomarker of response to immunotherapy in patients with lung cancer is lacking. Robust, adequately powered, observational and prospective clinical studies should continue to assess the association of TMB and other biomarkers with clinical outcomes of immunotherapy. We recommend that future studies assessing TMB adhere to standard reporting practices to enable comparison. The ongoing Friends of Cancer Research TMB Harmonization Project aims to provide a WES-based universal standard and to identify sources of variability once TMB scores from targeted panels have been aligned with the reference standard [112]. As further research is conducted with standardized TMB testing and reporting practices, a future meta-analysis may provide important evidence regarding the value of TMB testing in patients with lung cancer and other cancers.

In conclusion, these findings suggest that TMB may complement PD-L1 and dMMR/MSI testing in identifying patients among the lung cancer population who are likely to have good outcomes with immunotherapy. Robust, adequately powered, prospective and observational studies assessing TMB using standardized methodology are required to fully inform treatment decisions.

## SUPPLEMENTARY MATERIALS








