# Supplementary material for: Tumor mutational burden in lung cancer: a systematic literature review
Source: Oncotarget. 2019 Nov 12;10(61):6604–22. doi: 10.18632/oncotarget.27287 (PMC6859921; doi:10.18632/oncotarget.27287)
Supplement: Supplementary file 1 [file oncotarget-10-6604-s001.pdf]

Tumor mutational burden in lung cancer: a systematic literature review

SUPPLEMENTARY MATERIALS

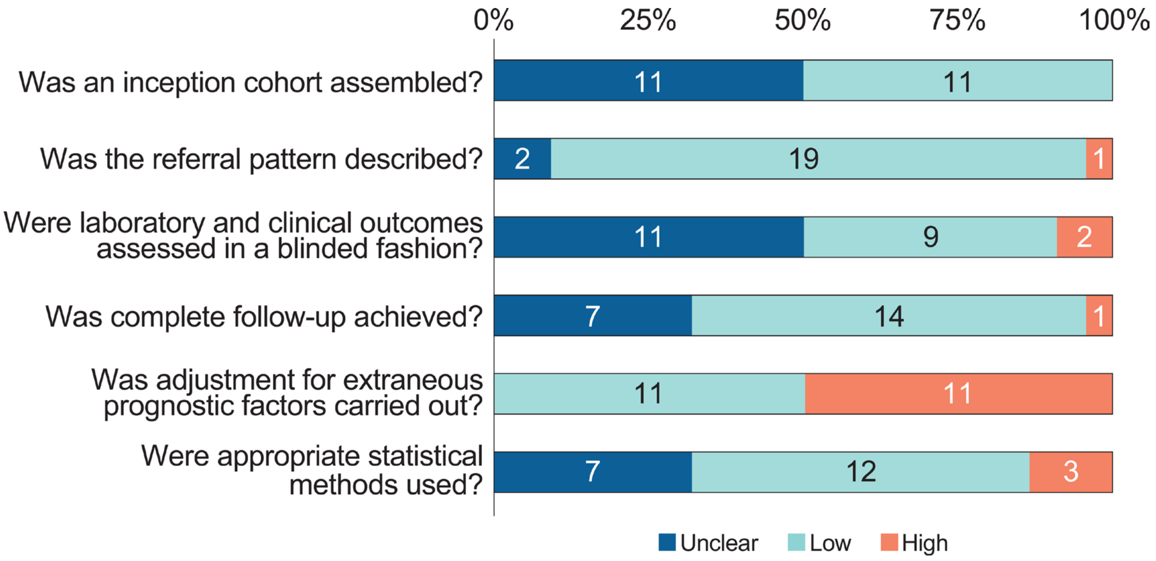

Supplementary Figure 1: Risk of bias results for clinical efficacy studies.

Supplementary Table 1: Full MEDLINE search strategy. See Supplementary Table 1

Supplementary Table 2: Full Emcare search strategy. See Supplementary Table 2

Supplementary Table 3: Full EMBASE search strategy

**EMBASE. com**<br><br>**Search Date: Wed April 10 (18:02:06 UTC) 2018**
((((('non small cell lung cancer'/mj OR 'large cell lung carcinoma'/mj OR 'lung adenocarcinoma'/mj OR 'squamous cell lung carcinoma'/mj OR 'lung cancer'/exp/mj) OR ((bronchial OR bronchus OR lung OR pleural OR pulmonary) NEAR/3 (adenocarcinoma* OR cancer OR cancers OR carcinom* OR carcinogenes* OR metasta* OR 'non small cell' OR 'large cell' OR 'squamous cell')): ti, ab, kw) AND ((((((tumor$ OR tumour$) NEAR/2 mutation*): ti, ab, kw) OR ((mutation* NEAR/2 (burden$ OR load$)): ti, ab, kw)) OR (landscape NEAR/2 mutation*): ti, ab, kw) OR ('monoclonal antibody'/mj OR ('monoclonal antibody mpdl 3280a': ti, ab, kw OR 'monoclonal antibody mpdl3280a': ti, ab, kw OR 'mpdl 3280a': ti, ab, kw OR mpdl3280a: ti, ab, kw OR 'rg 7446': ti, ab, kw OR rg7446: ti, ab, kw OR tecentriq: ti, ab, kw OR tecntriq: ti, ab, kw) OR (atezolizumab$: ti, ab, kw OR avelumab$: ti, ab, kw OR durvalumab$: ti, ab, kw OR pembrolizumab$: ti, ab, kw OR nivolumab$: ti, ab, kw OR ipilimumab$: ti, ab, kw) OR (bavencio: ti, ab, kw OR 'msb 0010682': ti, ab, kw OR 'msb 0010718c': ti, ab, kw OR 'msb 10682': ti, ab, kw OR 'msb 10718c': ti, ab, kw OR msb0010682: ti, ab, kw OR msb0010718c: ti, ab, kw OR 'msb10682': ti, ab, kw OR msb10718c: ti, ab, kw OR imfinzi: ti, ab, kw OR 'medi 4736': ti, ab, kw OR medi4736: ti, ab, kw OR keytruda: ti, ab, kw OR lambrolizumab: ti, ab, kw OR 'mk 3475': ti, ab, kw OR 'mk3475': ti, ab, kw OR 'bms 936558': ti, ab, kw OR bms936558: ti, ab, kw OR 'mdx 1106': ti, ab, kw OR mdx1106: ti, ab, kw OR 'ono 4538': ti, ab, kw OR ono4538: ti, ab, kw OR 'opdivo': ti, ab, kw OR 'bms 734016': ti, ab, kw OR 'bms734016': ti, ab, kw OR 'mdx 010': ti, ab, kw OR 'mdx 101': ti, ab, kw OR mdx010: ti, ab, kw OR mdx101: ti, ab, kw OR strentarga: ti, ab, kw OR yervoy: ti, ab, kw) OR ('atezolizumab'/de OR 'avelumab'/de OR 'durvalumab'/de OR 'pembrolizumab'/mj OR 'nivolumab'/mj OR 'ipilimumab'/mj)) OR ('cytotoxic t lymphocyte antigen 4'/mj OR ('antigen$ cd152': ti, ab, kw OR 'cd152 antigen$': ti, ab, kw OR 'ctla 4': ti, ab, kw OR 'cytotoxic t lymphocyte associated antigen 4': ti, ab, kw OR ctla4: ti, ab, kw)))) AND (('clinical study'/mj OR 'clinical trial'/mj OR 'controlled clinical trial'/mj OR 'controlled study'/mj OR 'major clinical study'/mj OR 'randomized controlled trial'/mj OR 'control group'/mj OR (((clinical OR randomi* OR controlled OR multicentre OR multicenter OR 'multi centre' OR 'multi center') NEAR/3 (study OR trial)): ti, ab) OR placebo: ab, ti OR 'head to head': ti, ab) OR 'panel study'/exp OR ('comparative study'/exp/mj OR 'controlled study'/exp/mj OR 'experimental study'/exp/mj OR 'observational study'/mj OR 'panel study'/mj OR 'quasi experimental study'/mj OR 'clinical study'/exp/mj) OR ('open label': ti, ab, kw OR 'open study': ti, ab, kw) OR (phase NEAR/2 (i OR ii OR iii OR iv OR '1' OR '2' OR '3' OR '4') NEAR/3 (study OR trial)): ti, ab, kw OR ((time OR times) NEAR/2 (duration$ OR frame OR frames OR period$ OR point$) NEAR/3 (over OR multiple OR three OR four OR five OR six OR seven OR eight OR nine OR ten OR eleven OR twelve OR month* OR hour$ OR day$ OR 'more than')): ti, ab, kw OR (before NEAR/3 after): ti, ab, kw OR ((before OR after) NEAR/5 during): ti, ab, kw OR cohort$: ti, ab, kw OR ('adverse event$': ti, ab, kw OR 'adverse effect$': ti, ab, kw) OR (analys*: ti, ab, kw OR design$: ti, ab, kw OR investigation$: ti, ab, kw OR study: ti, ab, kw OR studies: ti, ab, kw OR trial: ti, ab, kw OR trials: ti, ab, kw) OR ((case OR cases OR 'cba' OR control* OR historical OR crosssection* OR 'cross section*' OR crossection* OR epidemiolog* OR etiolog* OR followed OR 'follow-up' OR followup* OR longitudinal* OR retrospectiv* OR registry OR registries OR risk OR 'risk factor' OR prognostic OR observational* OR prospectiv*) NEAR/3 (study OR studies OR trial OR trials OR investigation* OR analys*)): ti, ab, kw) NOT (('animal'/exp OR 'invertebrate'/exp OR 'animal experiment'/exp OR 'animal model'/exp OR 'animal tissue'/exp OR 'animal cell'/exp OR 'nonhuman'/de) NOT (('animal'/exp OR 'invertebrate'/exp OR 'animal experiment'/exp OR 'animal model'/exp OR 'animal tissue'/exp OR 'animal cell'/exp OR 'nonhuman'/de) AND ('human'/exp OR 'human cell'/de)))) AND (2011: py OR 2012: py OR 2013: py OR 2014: py OR 2015: py OR 2016: py OR 2017: py OR 2018: py)) AND [english]/lim NOT ('conference abstract'/it OR 'conference review'/it) 1605

Supplementary Table 4: Measures reported by publications presenting efficacy, biomarkers or patient/disease characteristics data. See Supplementary Table 4

Supplementary Table 5: Publications reporting additional efficacy outcomes

	Main findings relating to TMB and respective outcome	*P* value
**DURABLE CLINICAL BENEFIT**		
Roszik 2016 [39]		
Pembrolizumab	DCB rate higher in patients with TMB-H (63%) vs TMB-L (0%)	NR
Hellmann 2017 [34]		
PD-L1 inhibitor +/− antiCTLA-4 therapy	TMB higher in patients with DCB (10.2 mut/Mb) vs no DCB (7.1 mut/Mb)	.02
Mahadevan 2017 [36]		
PD-1 inhibitors (*n* = 82), PD-L1 inhibitors (*n* = 5) other agents (*n* = 7)	TMB higher in patients with DCB (12 mut/Mb) than no DCB (8 mut/Mb)	.0027
Rizvi 2015 [1]		
Pembrolizumab (cohort 1)	TMB higher in patients with DCB (302 mut) than no DCB (148 mut)	.02
Pembrolizumab (cohort 2)	TMB higher in patients with DCB (244 mut) than no DCB (125 mut)	.04
**DISEASE CONTROL RATE**		
Singal 2017 [41]		
Nivolumab	DCR higher in patients with TMB-H (100%) vs TMB-L (62%)	.03
Hu 2018 [35]		
PD-1/PD-L1 inh5ibitors	DCR 100% in patients with TMB-H	NR
**DURATION OF TREATMENT**		
Singal 2017 [41]		
Nivolumab	DoT was longer in patients with TMB-H (7.5 months) vs TMB-L (4.6 months)	.001
Ross 2017 [45]		
Immune checkpoint inhibitors	DoT was longer in patients with TMB-H vs TMB-L (no values reported)	.001
**RECIST CRITERIA**		
Rozenblum 2017 [40]		
Pembrolizumab and nivolumab	Mean TMB in patients with:<br>–Partial response: 9.6 mut/Mb<br>–Stable disease 3.6 mut/Mb<br>–Progressive disease: 6.1 mut/Mb	NR

Abbreviations: DCB, durable clinical benefit; DoT, duration on therapy; mut/Mb, mutations per DNA megabase; PD-1, programmed cell death-1; PD-L1, programmed death ligand 1; TMB, tumor mutational burden; TMB-H, high TMB; TMB-L, low.
