# Supplementary material for: Tumor mutational burden in lung cancer: a systematic literature review
Source: Oncotarget. 2019 Nov 12;10(61):6604–22. doi: 10.18632/oncotarget.27287 (PMC6859921; doi:10.18632/oncotarget.27287)
Supplement: Supplementary file 2 [file oncotarget-10-6604-s002.docx]

**Supplementary Table 1:** **Full MEDLINE Search Strategy**

| Ovid MEDLINE® Epub Ahead of Print, In-Process & Other Non-Indexed Citations, Ovid MEDLINE® Daily and Ovid MEDLINE® < 1946 to January 22, 2018 | |
| --- | --- |
| 1 | Programmed Cell Death 1 Receptor/ (3141) |
| 2 | (pdcd1 ligand 1 or pdcd1lg1 protein* or programmed cell death 1 ligand 1 or programmed death 1 ligand 1 protein* or programmed death ligand 1 or protein pdcd1lg1 or pd1 or pd-1).ti,ab,kw,kf,rn. (8801) |
| 3 | (((b7 h1 or b7h1) adj2 (antigen* or protein*)) or b7 homolog 1 protein or cd274 antigen*).ti,ab,kw,kf,rn. (714) |
| 4 | (hPD-1 protein? or programmed death protein? or 1PD1 protein? or programmed cell death 1 protein? or CD279 antigen?).ti,ab,kw,kf,rn. (52) |
| 5 | or/1-4 [PD-1] (9597) |
| 6 | exp Antibodies, Monoclonal/ (209516) |
| 7 | (monoclonal antibody mpdl 3280a or monoclonal antibody mpdl3280a or mpdl 3280a or mpdl3280a or rg 7446 or rg7446 or tecentriq or tecntriq).ti,ab,kw,rn. (49) |
| 8 | (atezolizumab? or avelumab? or durvalumab? or pembrolizumab? or nivolumab? or ipilimumab?).ti,ab,kw,kf,rn. (4238) |
| 9 | (bavencio or "msb 0010682" or msb 0010718c or msb 10682 or msb 10718c or msb0010682 or msb0010718c or msb10682 or msb10718c or imfinzi or medi 4736 or medi4736 or keytruda or lambrolizumab or mk 3475 or mk3475 or bms 936558 or bms936558 or mdx 1106 or mdx1106 or ono 4538 or ono4538 or opdivo or bms 734016 or bms734016 or "mdx 010" or mdx 101 or mdx010 or mdx101 or strentarga or yervoy).ti,ab,kw,kf,rn. (244) |
| 10 | or/6-9 [Monoclonal Antibodies] (211679) |
| 11 | CTLA-4 Antigen/ (4708) |
| 12 | (antigen cd152 or cd152 antigen or ctla 4 or cytotoxic t lymphocyte associated antigen 4 or ctla4).ti,ab,kw,rn. (8461) |
| 13 | or/11-12 [CTLA-4 Antigen] (8461) |
| 14 | (((tumor? or tumour?) adj2 mutation$) or (mutation$ adj2 (burden? or load?))).ti,ab,kw,kf. (6009) |
| 15 | (landscape adj2 mutation*).ti,ab,kw,kf. (528) |
| 16 | TMB.ti,ab,kw,kf. (2203) |
| 17 | or/14-16 [TMB] (8661) |
| 18 | bronchial neoplasms/ or carcinoma, bronchogenic/ or carcinoma, non-small-cell lung/ or small cell lung carcinoma/ or exp lung neoplasms/ (205978) |
| 19 | ((bronchial or bronchus or lung or pleural or pulmonary) adj3 (adenocarcinoma$ or cancer or cancers or carcinom$ or carcinogenes$ or metastat$ or non small cell or large cell or squamous cell)).ti,ab,kw,kf. (178730) |
| 20 | exp pleural neoplasms/ (12838) |
| 21 | or/18-20 [Lung Cancer ] (272077) |
| 22 | (randomized controlled trial or controlled clinical trial).pt. or randomized.ab. or placebo.ab. or clinical trials as topic.sh. or randomly.ab. or trial.ti. (1132142) |
| 23 | exp animals/ not humans.sh. (4423824) |
| 24 | 22 not 23 [Cochrane RCT Filter 6.4.d Sens/Precision Maximizing] (1042591) |
| 25 | (animal? or beaver? or beef or bovine or breeding or bull or canine or castoris or cat or cattle or cats or chicken? or chimp$ or cow or dog or dogs or equine or feline? or foal or foals or fish or insect? horse or horses or livestock or mice or monkey? or mouse or murine or plant or plants or pork or porcine or protozoa? or purebred or rat or rats or rodent? or sheep or simian? or thoroughbred).ti. or veterinar$.ti,ab,kw,kf,hw. (2176512) |
| 26 | (clinical study or clinical trial or clinical trial phase i or clinical trial phase ii or clinical trial phase iii or clinical trial phase iv).pt. (547801) |
| 27 | (open label or open study).ti,ab,kw,kf. (38209) |
| 28 | (phase adj2 (i or ii or iii or iv or "1" or "2" or "3" or "4") adj3 (study or trial)).ti,ab,kw,kf. (60828) |
| 29 | (animal? or beaver? or beef or bovine or breeding or bull or canine or castoris or cat or cattle or cats or chicken? or chimp$ or cow or dog or dogs or equine or feline? or foal or foals or fish or insect? horse or horses or livestock or mice or monkey? or mouse or murine or plant or plants or pork or porcine or protozoa? or purebred or rat or rats or rodent? or sheep or simian? or thoroughbred).ti. or veterinar$.ti,ab,kw,kf,hw. (2176512) |
| 30 | (or/26-28) not (or/23,25) [Additional Trial Terms--Filter] (580491) |
| 31 | Observational Study/ (43503) |
| 32 | Cohort studies/ (217716) |
| 33 | Case-Control Studies/ (242598) |
| 34 | Follow-up Studies/ (583274) |
| 35 | exp Longitudinal Studies/ (112157) |
| 36 | Historically Controlled Studies/ (129) |
| 37 | Cross-sectional Studies/ (256446) |
| 38 | Epidemiological Studies/ or (Epidemiological methods/ and (196$ or 197$ or 198$).yr.) [Indexing for Epi studies prior to 1989] (18907) |
| 39 | "Controlled Before-after studies"/ or Interrupted Time Series Analysis/ (664) |
| 40 | Retrospective Studies/ (669453) |
| 41 | Prospective Studies/ (462882) |
| 42 | ((time or times) adj2 (duration? or frame or frames or period? or point?) adj3 (over or multiple or three or four or five or six or seven or eight or nine or ten or eleven or twelve or month$ or hour? or day? or "more than")).ti,ab,kw,kf. (44420) |
| 43 | (before adj3 after).ti,ab,kw,kf. (274313) |
| 44 | ((before or after) adj5 during).ti,ab,kw,kf. (193150) |
| 45 | cohort?.ti,ab,kw,kf. (443835) |
| 46 | (adverse event? or adverse effect? or ((case or cases or CBA or control$ or historical or cro??section$ or cross section$ or epidemiolog$ or etiolog$ or followed or followup? or "follow up" or ITS or longitudinal$ or long term or mortality or observational$ or prospectiv$ or retrospectiv$ or risk? or risk factor?) adj3 (analys?s or design? or investigation? or study or studies or trial or trials))).ti,ab,kw,kf. (2018119) |
| 47 | case series.ti,ab,kw,kf. (57708) |
| 48 | (or/31-47) not (or/23,25) [Observational Study Designs] (3513792) |
| 49 | or/24,30,48 [Methodological Filters-excluding animal studies] (4142784) |
| 50 | (2012$ or 2013$ or 2014$ or 2015$ or 2016$ or 2017$ or 2018$).yr,dp. (6575182) |
| 51 | (or/5,10,13,17) and 21 and 49 and 50 [RESULTS] (1426) |
| 52 | remove duplicates from 51 (1412) |
| 53 | (and/17,21,50) not 52 [TMB terms no filters] (291) |
| 54 | remove duplicates from 53 (289) |
