# Supplementary material for: Tumor mutational burden in lung cancer: a systematic literature review
Source: Oncotarget. 2019 Nov 12;10(61):6604–22. doi: 10.18632/oncotarget.27287 (PMC6859921; doi:10.18632/oncotarget.27287)
Supplement: Supplementary file 3 [file oncotarget-10-6604-s003.docx]

**Supplementary Table 2: Full Emcare Search Strategy**

| Database: Ovid Emcare <1995 to 2018 week 10>  TRIALS | |
| --- | --- |
| 1 | Programmed Cell Death 1 Receptor/ (1169) |
| 2 | (pdcd1 ligand 1 or pdcd1lg1 protein* or programmed cell death 1 ligand 1 or programmed death 1 ligand 1 protein* or programmed death ligand 1 or protein pdcd1lg1 or pd1 or pd-1).ti,ab,kw,kf,rn. (1725) |
| 3 | (((b7 h1 or b7h1) adj2 (antigen* or protein*)) or b7 homolog 1 protein or cd274 antigen*).ti,ab,kw,kf,rn. (6) |
| 4 | (hPD-1 protein? or programmed death protein? or 1PD1 protein? or programmed cell death 1 protein? or CD279 antigen?).ti,ab,kw,kf,rn. (13) |
| 5 | or/1-4 [PD-1] (2138) |
| 6 | *Antibodies, Monoclonal/ (2688) |
| 7 | atezolizumab/ or avelumab/ or durvalumab/ or pembrolizumab/ or nivolumab/ or ipilimumab/ (2557) |
| 8 | (monoclonal antibody mpdl 3280a or monoclonal antibody mpdl3280a or mpdl 3280a or mpdl3280a or rg 7446 or rg7446 or tecentriq or tecntriq).ti,ab,kw,rn. (14) |
| 9 | (atezolizumab? or avelumab? or durvalumab? or pembrolizumab? or nivolumab? or ipilimumab?).ti,ab,kw,kf,rn. (2587) |
| 10 | (bavencio or "msb 0010682" or msb 0010718c or msb 10682 or msb 10718c or msb0010682 or msb0010718c or msb10682 or msb10718c or imfinzi or medi 4736 or medi4736 or keytruda or lambrolizumab or mk 3475 or mk3475 or bms 936558 or bms936558 or mdx 1106 or mdx1106 or ono 4538 or ono4538 or opdivo or bms 734016 or bms734016 or "mdx 010" or mdx 101 or mdx010 or mdx101 or strentarga or yervoy).ti,ab,kw,kf,rn. (81) |
| 11 | or/7-10 [Monoclonal Antibodies] (2618) |
| 12 | or/6-10 [Monoclonal Antibodies] (5240) |
| 13 | cancer immunotherapy/ or immunotherap$.ti,ab,kw. (13221) |
| 14 | CTLA-4 Antigen/ (1621) |
| 15 | (antigen cd152 or cd152 antigen or ctla 4 or cytotoxic t lymphocyte associated antigen 4 or ctla4).ti,ab,kw,rn. (1096) |
| 16 | or/14-15 [CTLA-4 Antigen] (1958) |
| 17 | (((tumor? or tumour?) adj2 (mutation or mutational)) or ((mutation or mutational) adj2 (burden? or load?))).ti,ab,kw,kf. (472) |
| 18 | (landscape adj2 mutation*).ti,ab,kw,kf. (95) |
| 19 | TMB.ti,ab,kw,kf. (133) |
| 20 | or/17-19 [TMB] (685) |
| 21 | bronchial neoplasms/ or carcinoma, bronchogenic/ or carcinoma, non-small-cell lung/ or small cell lung carcinoma/ or exp *lung neoplasms/ (24275) |
| 22 | (((lung? or non-small-cell) adj2 (cancer? or tumo?r$ or carcinom$)) or NSCLC).ti,ab,kw. (35348) |
| 23 | exp pleural neoplasms/ (2180) |
| 24 | or/21-23 (43189) |
| 25 | cohort analysis/ (113369) |
| 26 | methodology/ or cross-sectional study/ (206998) |
| 27 | controlled study/ or exp case control study/ or exp controlled clinical trial/ or pretest posttest control group design/ (1154724) |
| 28 | Observational Study/ (49246) |
| 29 | Cohort studies/ (113369) |
| 30 | Case-Control Studies/ (19050) |
| 31 | Follow-up Studies/ (242758) |
| 32 | exp Longitudinal Studies/ (44036) |
| 33 | Cross-sectional Studies/ (35951) |
| 34 | "Controlled Before-after studies"/ or Interrupted Time Series Analysis/ (38387) |
| 35 | Retrospective Studies/ (85818) |
| 36 | Prospective Studies/ (84773) |
| 37 | ((time or times) adj2 (duration? or frame or frames or period? or point?) adj3 (over or multiple or three or four or five or six or seven or eight or nine or ten or eleven or twelve or month$ or hour? or day? or "more than")).ti,ab,kw,kf. (14934) |
| 38 | (before adj3 after).ti,ab,kw,kf. (83075) |
| 39 | ((before or after) adj5 during).ti,ab,kw,kf. (49898) |
| 40 | cohort?.ti,ab,kw,kf. (180988) |
| 41 | ((adverse event? or adverse effect? or AE or case or cases or CBA or cohort? or (control$ adj2 (case? or historical)) or cro??section$ or cross section$ or epidemiolog$ or etiolog$ or followed or followup? or "follow up" or ITS or longitudinal$ or long term or mortality or observational$ or prospectiv$ or retrospectiv$ or risk? or risk factor?) adj3 (analys?s or design? or investigation? or study or studies or trial or trials)).ti,ab,kw,kf. (602819) |
| 42 | case series.ti,ab,kw,kf. (21044) |
| 43 | observational study/ (49246) |
| 44 | (randomi?ed or placebo).ti,ab. or randomly.ab. or trial.ti. (334019) |
| 45 | clinical trials as topic/ (17150) |
| 46 | (animal? or beaver? or beef or bovine or breeding or bull or canine or castoris or cat or cattle or cats or chicken? or chimp$ or cow or dog or dogs or equine or feline? or foal or foals or fish or insect? horse or horses or livestock or mice or monkey? or mouse or murine or plant or plants or pork or porcine or protozoa? or purebred or rat or rats or rodent? or sheep or simian? or thoroughbred).ti,kw. or veterinar$.ti,ab,kw,hw. (168254) |
| 47 | or/25-45 (2024911) |
| 48 | (or/25-45) not 46 [Filter] (1916121) |
| 49 | limit 47 to human (1561963) |
| 50 | or/48-49 [Filter exc animal] (1932615) |
| 51 | 5 or 12 or 13 or 16 [Immunotherapies] (18765) |
| 52 | and/24,51 [Lung Cancer & Immunotherapies] (1229) |
| 53 | and/20,24 [Lung Cancer & TMB] (86) |
| 54 | and/50,52 (529) |
| 55 | limit 54 to (english language and yr="2012 - 2018") (420)  Annotation: Lung Cancer & Immunother & Filter |
| 56 | limit 53 to (english language and yr="2012-2018") (76)  Annotation: Lung Cancer & TMB |
| 57 | (mutation$ adj2 (burden? or landscape?)).ti,ab,kw. and 24 [No Filters] (31) |
| 58 | limit 57 to (english language and yr="2012-2018") (29) |
| 59 | 55 or 56 or 58 (488)  Annotation: Results to export |
| 60 | remove duplicates from 59 (487) |
