# Supplementary material for: Tumor mutational burden in lung cancer: a systematic literature review
Source: Oncotarget. 2019 Nov 12;10(61):6604–22. doi: 10.18632/oncotarget.27287 (PMC6859921; doi:10.18632/oncotarget.27287)
Supplement: Supplementary file 4 [file oncotarget-10-6604-s004.docx]

**Supplementary Table 4: Measures reported by publications presenting efficacy, biomarkers or patient/disease characteristics data**

|  | OS | PFS | ORR | DCB | DCR | DoT | PD-L1 | EGFR | *TP53* | Smoking | Cancer Type | Sex | Cancer Stage | Age |
| --- | --- | --- | --- | --- | --- | --- | --- | --- | --- | --- | --- | --- | --- | --- |
| Carbone 2017[10] | X | X | X |  |  |  | X |  |  |  |  |  |  |  |
| Chae 2018[56] |  |  |  |  |  |  |  |  |  | X |  |  |  |  |
| Chen 2017[46] |  |  |  |  |  |  |  | X |  |  |  |  |  |  |
| Choi 2017[27] | X* | X* |  |  |  |  | X |  |  |  |  |  | X |  |
| Davis 2017[28] | X |  |  |  |  |  |  |  |  | X |  |  |  |  |
| Dong 2017[47] |  |  |  |  |  |  |  | X |  |  |  |  |  |  |
| Gettinger 2017[29] | X |  | X |  |  |  |  |  |  |  |  |  |  |  |
| Goldberg 2017[48] |  |  |  |  |  |  | X |  |  |  |  |  |  |  |
| Goodman 2017[16] | X | X | X |  |  |  |  |  |  |  |  |  |  |  |
| Kowanetz 2017[30] | X | X | X |  |  |  |  |  |  |  |  |  |  |  |
| Haratani 2017[31] |  |  | X |  |  |  |  |  |  |  |  |  |  |  |
| Hellmann 2018[33] |  | X |  |  |  |  |  |  |  |  |  |  |  |  |
| Hellmann 2018[32] | X |  | X |  |  |  |  |  |  |  |  |  |  |  |
| Hellmann 2017[34] |  | X |  | X |  |  |  |  |  |  |  |  |  |  |
| Hu 2018[35] |  |  |  |  | X |  |  |  |  |  |  |  |  |  |
| Isaka 2017[66] |  |  |  |  |  |  |  |  |  |  | X |  |  |  |
| Kadara 2017[57] |  |  |  |  |  |  |  |  |  | X |  |  |  |  |
| Kim 2017[58] |  |  |  |  |  |  |  |  |  | X |  |  | X |  |
| Kojima 2017[67] |  |  |  |  |  |  |  |  |  |  | X |  |  |  |
| Liu 2018[49] |  |  |  |  |  |  | X |  |  |  |  |  |  |  |
| Lizotte 2016[59] |  |  |  |  |  |  |  |  |  | X |  |  |  |  |
| Mahadevan 2017[36] |  | X |  | X |  |  | X |  |  | X |  |  |  |  |
| Nakagomi 2018[50] |  |  |  |  |  |  | X |  |  |  |  |  |  |  |
| Ono 2017[60] |  |  |  |  |  |  |  |  |  | X | X |  |  | X |
| Owada 2017[51] |  |  |  |  |  |  |  | X | X | X | X | X |  |  |
| Park 2017[37] | X |  |  |  |  |  |  |  |  |  |  |  |  |  |
| Patel 2017[38] | X |  |  |  |  |  |  |  |  |  |  |  |  |  |
| Quek 2018[61] |  |  |  |  |  |  |  |  |  | X |  |  |  |  |
| Reck 2017[62] |  |  |  |  |  |  |  |  |  | X |  |  |  |  |
| Rizvi 2015[1] |  | X | X | X |  |  |  |  |  | X |  |  |  |  |
| Ross 2017[45] |  |  |  |  |  | X | X |  |  |  |  |  |  |  |
| Roszik 2016[39] |  | X | X | X |  |  |  |  |  |  |  |  |  |  |
| Rothberg 2017[52] |  |  |  |  |  |  |  |  | X |  |  |  |  |  |
| Rozenblum 2017[40] |  |  |  | X |  |  |  |  |  |  |  |  |  |  |
| Schabath 2017[53] |  |  |  |  |  |  | X |  |  |  |  |  |  |  |
| Schrock 2017[54] |  |  |  |  |  |  |  |  | X |  | X |  |  |  |
| Schrock 2016[63] |  |  |  |  |  |  |  |  |  | X |  |  |  |  |
| Senarthne 2018[55] |  |  |  |  |  |  | X |  |  |  |  |  |  |  |
| Shim 2015[64] |  |  |  |  |  |  |  |  |  | X |  |  |  |  |
| Singal 2017[41] | X |  | X |  | X | X |  |  |  |  |  |  |  |  |
| Wang 2017[42] |  | X* |  |  |  |  |  |  |  | X |  |  |  | X |
| Xiao 2016[43] | X* |  |  |  |  |  |  |  |  |  |  | X | X | X |
| Xiao 2017[65] |  |  |  |  |  |  |  |  |  | X |  | X | X | X |
| Yaghmour 2016[44] | X |  |  |  |  |  |  |  |  |  |  |  |  |  |
| Zhang 2016[68] |  |  |  |  |  |  |  |  |  |  |  |  |  | X |

*Did not include an immunotherapy arm.

Abbreviations: DCB, durable clinical benefit; DCR, disease control rate; DoT, duration of therapy; ORR, objective response rate; OS, overall survival; PD-L1, programmed death ligand 1; PFS, progression-free survival.
